# Atheroprotective Properties of *Costus spicatus* (Jacq.) Sw. in Female Rats

**DOI:** 10.3390/life11030212

**Published:** 2021-03-08

**Authors:** Bethânia Rosa Lorençone, Lucas Pires Guarnier, Rhanany Alan Calloi Palozi, Paulo Vitor Moreira Romão, Aline Aparecida Macedo Marques, Lislaine Maria Klider, Roosevelt Isaias Carvalho Souza, Ariany Carvalho dos Santos, Cleide Adriane Signor Tirloni, Nadla Soares Cassemiro, Denise Brentan Silva, Jane Manfron Budel, Arquimedes Gasparotto Junior

**Affiliations:** 1 Laboratory of Cardiovascular Pharmacology (LaFaC), Faculty of Health Sciences, Federal University of Grande Dourados, Dourados 79.804-970, MS, Brazil; bethania.lorencone041@academico.ufgd.edu.br (B.R.L.); lucas.guarnier403@academico.ufgd.edu.br (L.P.G.); rhanany.palozi063@academico.ufgd.edu.br (R.A.C.P.); paulo.romao054@academico.ufgd.edu.br (P.V.M.R.); aline.marques391@academico.ufgd.edu.br (A.A.M.M.); RooseveltSouza@ufgd.edu.br (R.I.C.S.); ArianySantos@ufgd.edu.br (A.C.d.S.); cleidetirloni@ufgd.edu.br (C.A.S.T.); 2 Postgraduate Program in Pharmaceutical Sciences, State University of Ponta Grossa, Ponta Grossa 84.030-900, PR, Brazil; lislaine.klider@ufpr.br (L.M.K.); jane@uepg.br (J.M.B.); 3 Laboratory of Natural Products and Mass Spectrometry (LaPNEM), Faculty of Pharmaceutical Sciences, Food and Nutrition (FACFAN), Federal University of Mato Grosso do Sul, Campo Grande 79.070-900, MS, Brazil; nadla.cassemiro@ufms.br (N.S.C.); denise.brentan@ufms.br (D.B.S.)

**Keywords:** antioxidant, anti-inflammatory, Costaceae, dyslipidemia, flavonoids

## Abstract

Background: *Costus spicatus* (Jacq.) Sw. is a medicinal species frequently prescribed for the treatment of cardiovascular diseases. This study aims to evaluate the effects of this species against the development of atherosclerosis. Methods: First, an anatomical study of the *C. spicatus* leaves was performed. Then, the extract (ESCS) was obtained and submitted to phytochemical analysis. Female rats were treated with a single dose of ESCS (2000 mg/kg) to assess acute toxicity. Other groups of female rats received an atherogenic diet for 60 days. After 30 days, the animals were treated orally with ESCS (30 and 300 mg/kg), rosuvastatin (5 mg/kg), or vehicle once daily for 30 days. Serum lipids oxidized low-density lipoprotein, soluble adhesion molecules, interleukins 1β and 6, and markers of renal and liver function were measured. Renal function, blood pressure, electrocardiography, and vascular reactivity were also evaluated. Arteries, heart, liver, and kidney were also collected to evaluate the tissue redox state and histopathological analysis. Results: Prolonged treatment with ESCS induces significant hypolipidemic and antioxidant effects, that prevent endothelial dysfunction and modulated the local inflammatory process, reducing the evolution of the atherosclerotic disease. Conclusions: This study provides a scientific basis for the popular use of *C. spicatus* for the treatment of atherosclerosis.

## 1. Introduction

Plants are an important source of biologically active molecules, many of which are models for the synthesis of a large number of drugs [1]. Herbal medicine has been a common practice for thousands of years, but the search for active constituents in the medicinal plants started in the 19th century [2]. Currently, many pharmaceutical companies have expressed renewed interest in natural products as sources of new therapies [3].

The Brazilian population has a long tradition of using medicinal plants for the treatment of different acute and chronic diseases. *Costus spicatus* (Jacq.) Sw. (Costaceae) is a Brazilian medicinal species popularly known as “cana-do-brejo”, “cana-do-mato”, “cana-roxa”, and “cana-de-macaco”. It is a perennial plant whose aerial parts can reach 1–2 m in height. It is native mainly to Atlantic Forest and Amazon regions [4], and is widely used in home gardens due to the yellow flowers and red bracts. In traditional medicine, the aqueous extract of the rhizomes, stems, and leaves are used for the treatment of nephrolithiasis, urinary tract infections, and as a diuretic and depurative [4,5]. Furthermore, a recent ethnobotanical study carried out in the region of Grande Dourados, located in the state of Mato Grosso do Sul (Brazil), showed that an infusion of the leaves of *C. spicatus* is frequently prescribed by local healers for the treatment of cardiovascular diseases [6].

Several secondary metabolites were identified in different preparations obtained from *C. spicatus*, including flavonoids, saponins, tannins, alkaloids, polysaccharides, and triterpenes [4,7,8,9,10,11,12]. Preclinical pharmacological studies have reported analgesic, anti-inflammatory [4,12,13,14], nephroprotective and antilithiasic [9], antidiabetic [15], and antifungal activities [16].

Despite folk medicine suggest possible cardioprotective activities for the *C. spicatus,* scientific data on its hypolipidemic and anti-atherosclerotic effects remain unknown. In addition, although females are known to have specific cardiovascular characteristics, the treatments that are specifically directed toward this gender are nonexistent [17]. Therefore, the aim of the present study was to evaluate the cardioprotective effects of the purified aqueous extract obtained from *C. spicatus* leaves in an experimental model of atherosclerosis in female rats. Moreover, a detailed botanical, phytochemical, and toxicological study were also performed.

## 2. Materials and Methods

### 2.1. Plant Material

*Costus spicatus* leaves were collected in March 2019 in the municipality of Dourados, Mato Grosso do Sul, Brazil (22°12′21.2″ S, 54°55′08.0″ W). All procedures were authorized by the National System of Genetic Resource Management and Associated Traditional Knowledge (SisGen no. A8DF115). The plant was authenticated by Dr. Zefa Valdivina Pereira and deposited in the herbarium of the Federal University of Grande Dourados (UFGD; no. 5645).

### 2.2. Anatomical Study

Part of the plant material was fixed in a 70% formalin solution, acetic acid, and ethanol [18]. After 3 days, the leaves were stored in 70% ethanol (*v*/*v*; [19]). Transverse free-hand sections and bleaching of the leaves were performed according to Santos et al. [20]. Histochemical tests were performed using leaves with the following reagents: phloroglucinol/HCl [21], Sudan III [22], 2% ferric chloride, 10% potassium dichromate [18], 1% iodine solution [19], and 1% methylene blue [23]. Photomicrographs were acquired using an Olympus CX31 microscope equipped with an Olympus C-7070 digital camera. Scanning electron microscopy (SEM) and EDS of the leaves of *C. spicatus* were detailed in a previous report [24].

### 2.3. Phytochemical Study

#### Extractive Procedure

*C. spicatus* leaves were dried for 7 days (40 °C oven). After drying, the leaves were ground in a hammer mill. The infusion was produced as recommended by popular use [6] by adding 200 mL of pre-boiling water for each 2.5 g of dried and ground leaves. Extraction was performed until room temperature was reached (~5 h). Then, the infusion was treated with 3 volumes of ethanol (99.8%; Merck KGaA, Darmstadt, Germany). The precipitate was eliminated, resulting in an ethanol-soluble fraction (ESCS). Ethanol was totally removed by rotary evaporation, and the extract was freeze-dried (4.70% yield). Liquid chromatography coupled with a diode array detector and mass spectrometry (LC-DAD-MS).

The LC-DAD-MS analyses were performed using a Shimadzu LC-20AD UFLC chromatograph and mass spectrometer with an electrospray ion source and quadrupole-time-of-flight analyzer (microTOF III, BrukerDaltonics, Billerica, MA, USA). The chromatographic parameters were the same as those that were applied by Moreno et al. [9] using a Kinetex C18 column (Phenomenex, 2.6 μm, 150 × 2.1 mm) and a mobile phase that consisted of acetonitrile and ultrapure water to which 0.1% formic acid (*v*/*v*) was added. The MS and MS/MS analyses were performed in negative and positive ion modes. 

The ESCS was solubilized in methanol and water (6:4, *v*/*v*) and underwent liquid-liquid extraction with dichloromethane and ethyl acetate. Dichloromethane, ethyl acetate, and hydromethanol fractions were prepared in methanol and water (7:3, *v*/*v*) at a concentration of 1 mg/mL and filtered (filter Millex 0.22 µm, PTFE, Merck Millipore, Burlington, Massachusetts, EUA). Then, 1 μL of each sample was injected into the chromatographic system.

### 2.4. Animals

Female Wistar rats (12 weeks old; 210–250 g) were obtained from the Federal University of Grande Dourados (UFGD). Animals were housed in a temperature- and light-controlled room (12-h light/dark cycle; 22 ± 2 °C) with free access to food and water. Before the onset of all experiments, animals were left for ten days to acclimatize to laboratory conditions. All procedures involving animals were previously approved by the Ethics Committee in Animal Experimentation from UFGD (protocol: 13/2018).

### 2.5. Toxicological Study

#### Acute Oral Toxicity

Acute toxicity was evaluated in female rats according to protocol 425 established by the Organization for Economic Co-operation and Development (OECD) in 2008. After 8 h of fasting, 2000 mg/kg ESCS was administered to female rats (n = 5) by oral gavage. The control group (n = 5) was treated with filtered water (1 mL/100 g). After 1 h of treatment, the animals were fed. The evidence of death or toxicity signs were monitored by behavioral and clinical parameters during the first 8 h after the treatments, and daily for 14 days. Bodyweight gain and food and water consumption were daily measured and registered. The following parameters of the Hippocratic screening described by Malone and Robichaud [25] were observed: conscious state, activity, and coordination of motor system, muscle toning, effects on the central and autonomic nervous system, and corneal and headset reflexes. On the 15th day, the animals were euthanized by inhalation of isoflurane (50–70%) in the saturation chamber, followed by exsanguination. Reproductive (uterus and ovaries) and vital organs (heart, lung, liver, spleen, and kidneys) were removed to the determination of relative weight (absolute weight organ × 100/body weight from each animal) and macroscopically examined.

### 2.6. Pharmacological Study

The doses of the ESCS were defined from the study of Coelho et al. [6]. In this work, it has been described that the most commonly reported preparation is the use of 200 mL of pre-boiling water directly poured into an amount of crushed plant equivalent to a ‘closed hand’ (~2.5 g). By dividing 2.5 g by the average weight of a human adult (70 kg), we will have an approximate amount of 30 mg/kg. Thus, we chose to use a dose of 30 mg/kg and, from it, a 10-fold higher dose, i.e., 300 mg/kg.

Female rats were initially placed in five experimental groups (n = 8/group): naïve (animals treated orally with the vehicle (filtered water; 0.1 mL/100 g) and fed with standard feed), negative control (NC; animals treated orally with the vehicle (filtered water; 0,1 mL/100 g) and fed with atherogenic diet (AD)), ESCS 30 (animals treated orally with the ESCS 30 mg/kg and fed with AD), ESCS 300 (animals treated orally with the ESCS 300 mg/kg and fed with AD), and ROSU (animals treated orally with the rosuvastatin 5 mg/kg and fed with AD). Animals in the NC, ESCS (30 and 300), and ROSU groups received daily methimazole (2 mg/kg: by oral route) and four injections, every 2 days, of 175,000 IU/kg cholecalciferol (by intraperitoneal route) [26]. Initial and final body weight were noted. The AD was prepared as described by Guarnier et al. [26] with the following composition: 64.4% standard diet, 5% lard, 5% sucrose, 5% hydrogenated fat, 0.5% cholesterol, 0.1% sodium cholate, and 20% egg powder. The feed was provided *ad libitum* for 60 days. The pharmacological treatments started on the 31^st^ day of the diet and were carried out by oral route (once a day) for another 30 days.

### 2.7. Experimental Procedures

#### 2.7.1. Renal Function Assay

Renal function was evaluated according to Gasparotto et al. [27]. At the end of the treatments, all animals received 5 mL/100 g of physiological saline solution (0.9% NaCl) by oral route to impose salt and water balance. Afterward, the animals were placed in metabolic cages with free access to food and water. Urine samples were collected in graduated cylinders, and urine volumes were recorded by 24 h. The results are expressed as mL/100 g of body weight. The pH and density were determined using a digital pH meter (Q400MT; Quimis Instruments, Diadema, Brazil) and a handheld refractometer (NO107; Nova Instruments, Piracicaba, Brazil), respectively. Urinary sodium, calcium, potassium, magnesium, chloride, and creatinine levels were quantified using an automated biochemical analyzer (COBAS INTEGRA 400 plus; Roche, Basel, Switzerland).

#### 2.7.2. Electrocardiography

After urine collection, the animals were anesthetized with ketamine (100 mg/kg) plus xylazine (20 mg/kg). Electrocardiography (ECG) was performed according to Guarnier et al. [26]. The electrodes were placed on the animal’s limbs. P, Q, R, and S waves and PR, QRS, QT, and QTC segments were recorded using an ECG recorder (WinCardio, Micromed, Brasilia, Brazil).

#### 2.7.3. Blood Pressure Measurement

After recording the ECG, and still under anesthesia, mean arterial pressure (MAP), systolic (SBP) and diastolic blood pressure (DBP), and heart rate (HR) were measured according to Gasparotto Junior et al. [28]. After heparin administration (30 IU; subcutaneously), the left carotid artery was cannulated and connected to a PowerLab recording system (Chart 7.1 software; ADInstruments, Castle Hill, Australia). Systolic blood pressure, DBP, MAP, and HR were recorded for 20 min. The results are presented as the average of the last 5 min recorded.

#### 2.7.4. Biochemical Analysis

After the arterial pressure measurements, blood samples were collected from the previously cannulated carotid artery. To obtain serum, the samples were centrifuged at 1500 g for 10 min. Serum total protein, albumin, globulin, alanine aminotransferase (ALT), alkaline phosphatase (AP), aspartate aminotransferase (AST), direct bilirubin (DB), indirect bilirubin (IB), total bilirubin (TB), creatinine, urea, triglycerides (TG), low-density lipoprotein cholesterol (LDL-C), high-density lipoprotein cholesterol (HDL-C), very-low-density lipoprotein cholesterol (VLDL-C), total cholesterol (TC), lactate dehydrogenase (LDH), sodium, calcium, potassium, magnesium, and chloride levels were quantified using an automated biochemical analyzer (COBAS INTEGRA 400 plus; Roche). Serum oxidized LDL (ox-LDL), soluble vascular cell adhesion molecule-1 (sVCAM-1), soluble intercellular adhesion molecule-1 (sICAM-1), interleukin 1-beta (IL-1β), and IL-6 levels were measured using an enzyme-linked immunosorbent assay kit (BD Biosciences, San Diego, CA, USA).

#### 2.7.5. Mesenteric Vascular Reactivity

The mesenteric vascular bed (MVB) was isolated according to previously described methods [29]. The superior mesenteric artery was cannulated and gently flushed with 0.5 mL of physiological saline solution (PSS; 119 mM NaCl, 25.0 mM NaHCO_3_, 11.1 mM C_6_H_12_O6, 4.7 mM KCl, 2.4 mM CaCl_2_, 1.2 mM MgSO_4_, 1.2 mM KH_2_PO_4_, and 0.03 mM C_10_H_16_N_2_O_8_) containing 250 IU/mL heparin to prevent blood clotting. After removal of the entire intestine, including the vascular bed, 10 mL of PSS was perfused through the superior mesenteric artery, and the mesenteric arterial vascular bed was separated from the intestine. The four main arterial branches from the superior mesenteric trunk running to the terminal ileum were perfused, and all other branches of the superior mesenteric vascular bed were tied off. The isolated mesenteric vascular bed was then placed in a water-jacketed organ bath maintained at 37 °C and perfused with PSS gassed with 95% O2/5% CO_2_, 37 °C at a constant flow rate of 4 mL/min through a peristaltic pump. After the setup in the perfusion apparatus, preparation was allowed to equilibrate for 30 to 45 min, and its viability was checked by a bolus injection of KCl (120 mmol). Then, four doses of phenylephrine (Phe; 1, 3, 10, and 30 nmol, 10–30 µL) were administered. After another 30 min of stabilization, 3 µM Phe was added to the PSS to induce a prolonged increase in perfusion pressure. The vascular reactivity to acetylcholine (ACh; 1, 3, 10, and 30 pmol) and sodium nitroprusside (SNP; 0.1, 0.3, 1, and 3 pmol) were also evaluated. The bolus injections of all tested substances were made at a final volume of 10 or 30 µL through access close to the vascular preparation. Responses started 10 s after administration. Changes in the perfusion pressure (mm Hg) were measured by a pressure transducer connected to an acquisition system (PowerLab^®^), and its application program (Chart, v 7.1; both from ADI Instruments, Castle Hill, Australia).

#### 2.7.6. Tissue Redox Status

After euthanasia, samples of the heart, right kidney, and aorta artery were sectioned and homogenized in phosphate buffer (0.1 M, pH 6.5). The homogenate was centrifuged at 5000 g for 20 min at 4 °C, and different dilutions were obtained. Superoxide dismutase (SOD) assay was performed according to Marklund and Marklund [30]. Lipoperoxidation (LPO) was also measured using a previously described method [31]. The results are expressed as U of SOD/g of tissue for SOD and mmol hydroperoxide/g of tissue for LPO levels.

#### 2.7.7. Histopathological and Morphometric Analyses

Samples of the heart ventricles, subclavian and carotid arteries, liver, and right kidney were dehydrated in alcohol, cleared with xylene, embedded in paraffin, and sectioned at a 5 mm thickness. The samples were stained with hematoxylin-eosin and orcein (only arteries) and examined under a light microscope. The right and left ventricles, interventricular septum, and intima layer of the subclavian and carotid arteries were measured using a caliper. For each animal, we assembled several blades with the entire tissue studied. For each blade, six lines of division were established. For each line were performed six measurements, and one mean for each slide was obtained. When all the slides were evaluated, a new mean for each animal was determined. The area of the microscope was adjusted using an ocular micrometer coupled with the 100X objective, determining the size of the field per μm. All images were obtained and evaluated by Motic Images Plus 2.0 software.

### 2.8. Statistical Analysis

The results are expressed as the mean ± standard error of the mean (SEM). Statistical analyses were performed using GraphPad Prism software (version 8.4.3). Differences between groups were evaluated using analysis of variance (ANOVA), followed by Bonferroni post hoc test. Values of *p* < 0.05 were considered statistically significant.

## 3. Results

### 3.1. Anatomical Study

**Figure 1:**
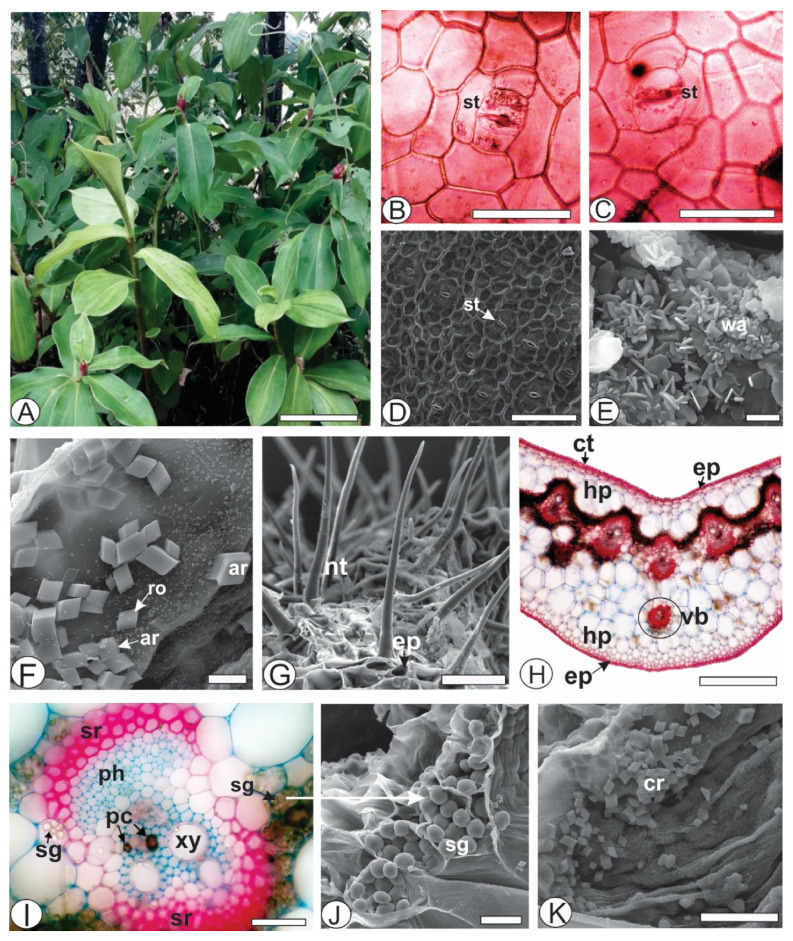
Morpho-anatomy of *Costus spicatus* (Jacq.) Sw. leaves [(**B**,**C**,**H**,**I**): light microscopy; (**D**–**G**,**J**,**K**): FESEM]. (**B**–**G**) Frontal view. (**H**–**K**) Transversal sections. [ar, arrowhead-shaped crystal; cr, sand crystal; ct, cuticle; ep, epidermis; hp, hypodermis; nt, non-glandular trichome; pc, phenolic compounds; ph, phloem; ro, rhombohedral crystal; sg, starch grain; sr, sclerenchyma; st, stomata; vb, vascular bundle; xy, xylem; wa, epicuticular wax]. Scale bar: (**A**) = 20cm; (**H**) = 500 µm; (**D**) = 100 µm; (**B**,**C**,**G**,**I**) = 50 µm; (**J**,**K**) = 20 µm; (**F**) = 5 µm; (**E**) = 2 µm.

The frontal view of *Costus spicatus* (Figure 1A) presented epidermal cells with straight to slightly wavy anticlinal walls (Figure 1B,C), prominently raised above the leaf surface. The external periclinal walls had a slightly striated cuticle (Figure 1D) and epicuticular waxes (Figure 1E). The stomata were tetracytic (Figure 1B,C), and the leaves were anfi-hypostomatic.

Crystal sand in different shapes, such as rhombohedral and arrowhead-shaped, was found externally on the adaxial side of the leaf (Figure 1F). The energy-dispersive X-ray spectroscopy (EDS) spectra of the crystals showed peaks of calcium (16.96%), carbon (35.24%), and oxygen (47.81%). The occurrence of these elements indicated that the crystals were formed by calcium oxalate. This characteristic was not previously mentioned for the genus and thus helps with species identification. These crystals on the epidermis are uncommon but have been found in some genus, such as *Mollinedia* [32], *Piper* [20], and *Talinum* [33]. Several simple non-glandular trichomes with a thick wall were observed, especially on the abaxial side (Figure 1G).

In the cross-section, the epidermis was unlayered (Figure 1G,H). Beneath the epidermis, two to three layers of rounded and voluminous hypodermis cells were found on both sides (Figure 1H). The mesophyll was dorsiventral and formed by one to two layers of palisade parenchyma, and two to three layers of spongy parenchyma. The mesophyll was divided into two regions that were traversed by a strand of fibers and collateral vascular bundles that were localized side by side. Sclerenchymatous fiber caps were adjoined to the phloem and xylem in bigger bundles (Figure 1H,I). Sclerenchymatous cells frequently continuously surrounded the minor vascular bundles (Figure 1H). Several starch grains were present close to the bundles (Figure 1I,J). Crystal sand was also observed in the hypodermis (Figure 1K) and palisade parenchyma. Additionally, the midrib had a concave-convex shape, and a line of vascular bundles in the middle and one isolated vascular bundle in the inferior portion of the mesophyll was observed (Figure 1H).

**Figure 2:**
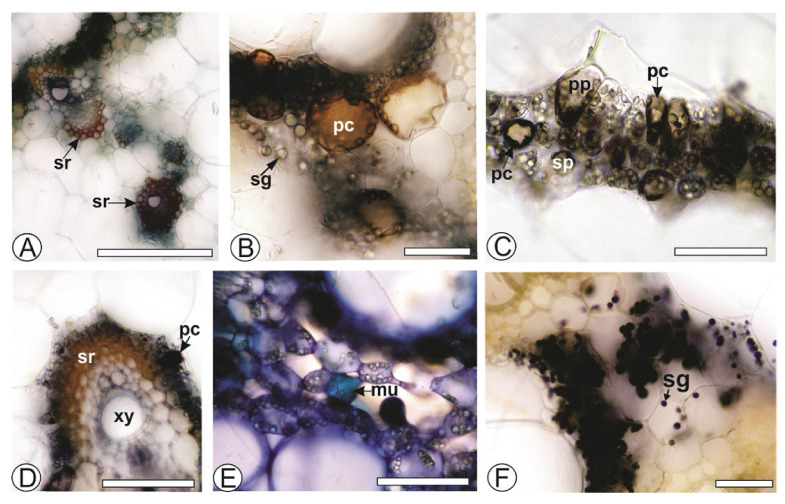
Histochemical tests of *Costus spicatus* (Jacq.) Sw. Cross-section of leaves. (**A**) Sclerenchyma in reaction with phloroglucinol/HCl; (**B**) Phenolic compounds in positive reaction with potassium dichromate; (**C**,**D**) Phenolic compounds in reaction with ferric chloride; (**E**) Mucilaginous cell in reaction with methylene blue; (**F**) Starch grains in positive reaction with iodine solution. (mu, mucilaginous cell; pc, phenolic compounds; pp, palisade parenchyma; sg, starch grains; sp, spongy parenchyma; sr, sclerenchyma; xy, xylem). Scale bar: (**A**) = 200 µm; (**B**–**F**) = 50 µm.

**Figure 3:**
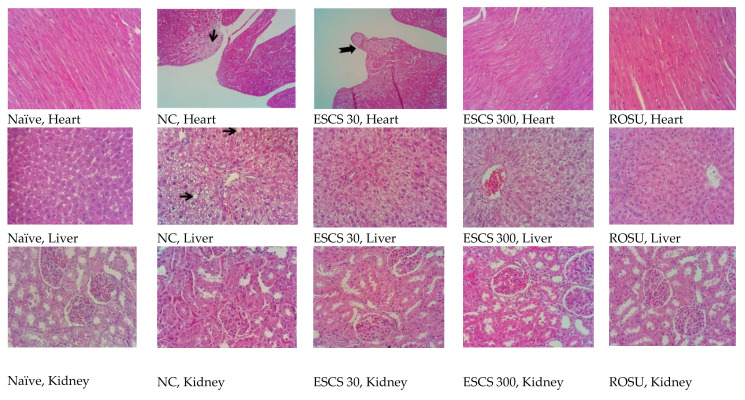
Representative cross-sections of the liver, heart, and kidney in the naïve, NC, ESCS (30 and 300 mg/kg), and ROSU groups. Black arrows indicate cardiac fibrosis and lipid macrovesicles in the liver. Hematoxylin-eosin staining (40×). NC, negative control group; ROSU, rosuvastatin.

The histochemical tests showed lignified components in the sclerenchyma (Figure 2A) and xylem. Phenolic compounds were observed in the cells of palisade and spongy parenchyma (Figure 2B,D) and reacted with potassium dichromate (Figure 2B) and ferric chloride (Figure 2C,D). Phenolic idioblasts were also observed in the phloem and xylem (Figure 1D). A few mucilaginous cells were found in the spongy parenchyma (Figure 3E), with several starch grains (Figure 2F).

### 3.2. Chemical Constituents of the Ethanol-Soluble Fraction of C. Spicatus (ESCS)

The ESCS was fractionated by liquid-liquid extraction, and dichloromethane, ethyl acetate, and hydromethanol fractions were analyzed by high-performance liquid chromatography coupled with diode-array detection and electrospray ionization tandem mass spectrometry (LC-DAD-MS). The identification of compounds was based on ultraviolet (UV), MS, and MS/MS spectral data compared with data in the literature.

In a previous study [9], the ESCS was analyzed by LC-DAD, and 25 compounds were annotated. In the present study, we identified 54 compounds (Table 1). Compounds 1–2, 4, 6–10, 13–14, 18–20, 24–28, 30, 33, 35, 37, and 41–42 were previously described [9]. These compounds included organic acids (pentonic acid {1} and citric acid {4}), phenylpropanoid derivative (6), glycosylated flavonoids (6,8-di-C-hexosyl apigenin {7}, C-hexosyl C-pentosyl apigenin {8–10 and 13–14}, rutin {18}, O-hexosyl quercetin {19}, C-hexosyl C-deoxyhexosyl apigenin {20}, O-pentosyl quercetin {24}, O-hexosyl-deoxyhexosyl luteolin {25}, O-hexosyl-deoxyhexosyl O-methyl quercetin {26 and 30}, O-hexosyl luteolin {27}, O-deoxyhexosyl quercetin {28}, O-hexosyl O-methyl quercetin {33}, O-pentosyl O-methyl quercetin {35}, and O-deoxyhexosyl luteolin {37}), and steroidal saponins (41 and 42).

Peak 4 revealed absorption bands at wavelengths of 268, 277, and 288 nm, and it’s protonated ion at m/z 205.0971 (C11H13N2O2+) yielded the fragment ion m/z 188 from the loss of 17 u (NH3). Compound 4 was putatively identified as tryptophan [34]. Peaks 15, 23, 29, 36, and 44 showed UV spectra (λmax = 260 and 350 nm) that were similar to flavonols, and peaks 21–22, 31–32, and 34 were identical to flavones (λmax = 265 and 330 nm; [9]). These compounds revealed losses of 162, 146, and 132 u, with the exception of peak 44 that is a non-glycosylated flavonol, confirming the presence of hexosyl, deoxyhexosyl, and pentosyl substituents, respectively. The metabolites 15/23 and 21–22/31–32/34 revealed aglycones at m/z 303 and 287 [aglycone+H]+. These data, together with characteristic fragment ions from the cleavage of pyran ring (C ring), suggested the genins quercetin and luteolin. 

Additionally, aglycon ions from 29, 36, and 44 revealed losses of a methyl radical (•CH3, 15 u), suggesting O-methyl quercetin and O-methyl kaempferol [9,35,36,37]. These compounds were annotated as O-hexosyl-deoxyhexosyl quercetin (15), O-deoxyhexosyl-hexosyl luteolin (21, 22), O-pentosyl quercetin (23), O-hexosyl O-methyl quercetin (29), O-pentosyl luteolin (31, 34), O-pentosyl-deoxyhexosyl luteolin (32), O-hexosyl O-methyl kaempferol (36), and O-methyl kaempferol (44).

Compounds 41–42 and 47–49 did not show UV absorption. From its ions ([M+H]+ and [M-H]-) were observed consecutive sugar losses, such as 162, 146, and 132 u, which confirmed hexosyl, deoxyhexosyl, and pentosyl groups [38]. Moreover, fragment ions m/z 415 and 433 were observed for 41/42 and 47/49, and they are correspondent to molecular formulas C27H42O3 and C27H46O4. All of the spectral data suggested steroidal saponins, which have been reported for *C. spicatus* [11,39].

### 3.3. Toxicological Study

#### Acute Oral Toxicity

No deaths were observed until the 15th day of the experiment, suggesting an LD50 above 2000 mg/kg. Similarly, no significant clinical or behavioral alterations were found in any of the experimental groups. The organs’ relative weights and histopathological analyses did not show any changes suggestive of toxicity that could be induced by ESCS or vehicle.

### 3.4. Pharmacological Study

#### 3.4.1. Body Weight Gain

The initial and final body weight did not show any statistically significant changes compared to the different experimental groups (data not shown).

#### 3.4.2. Renal Function

The effects of prolonged oral treatment with the ESCS (30 and 300 mg/kg) and rosuvastatin (ROSU) on urinary parameters are presented in Table 2. All animals that were fed the AD and did not receive any treatment exhibited a significant reduction of pH, density, and urinary chloride excretion when compared to naïve rats. The treatment with ESCS (30 and 300 mg/kg) or ROSU was able to prevent all these changes, maintaining urinary values similar to those obtained in naïve animals. Moreover, ESCS (30 and 300 mg/kg) treatment resulted in a greater elimination of urine, with values statistically higher than those obtained in animals of the naïve, NC, or ROSU groups.

#### 3.4.3. Electrocardiographic Parameters

No statistically significant electrocardiographic changes were observed between the naïve, NC, ESCS (30 or 300 mg/kg), or ROSU groups (Table 3).

#### 3.4.4. Blood Pressure and Heart Rate

None of the experimental groups showed any significant change in the values of MAP, SBP, DBP, or HR (Table 4).

#### 3.4.5. Biochemical Analyses

The effects of prolonged oral treatment with the ESCS (30 and 300 mg/kg) and ROSU on biochemical parameters are presented in Table 5. Serum levels of TG, TC, LDL-C, VLDL-C, and ox-LDL were significantly increased in the NC group when compared to the naïve group. The female rats that were treated with the ESCS (30 and 300 mg/kg) or ROSU exhibited a significant reduction in serum TG, TC, LDL-C, VLDL-C, and ox-LDL levels when compared to the NC group.

The levels of sVCAM-1, sICAM-1, IL-6, and IL-1β levels were significantly higher in NC rats than in naïve rats. Prolonged treatment with the ESCS (30 and 300 mg/kg) or ROSU significantly reduced sVCAM-1, sICAM-1, IL-6, and IL-1β levels when compared to NC rats. Creatinine, urea, AST, and ALT levels showed no statistically significant changes. Serum total protein, albumin, globulin, AP, LDH, IB, DB, TB, sodium, calcium, potassium, magnesium, and chloride were also not changed in any of the experimental groups (data not shown).

#### 3.4.6. Mesenteric Vascular Reactivity

Phenylephrine, ACh, and NPS reactivity on isolated and perfused MVBs are presented in Table 6. The NC group exhibited higher Phe reactivity in MVBs at 10 and 30 nmol when compared to naïve rats. The prolonged treatment with ESCS (at doses of 30 and 300 mg/kg) was able to prevent this change, maintaining values close to naïve animals. Although reactivity to Phe at the dose of 10 nmol was normalized by treatment with ROSU, when a dose of 30 nmol was administered to MVBs from ROSU-treated animals, the reactivity was significantly higher than that observed in naïve or ESCS-treated rats. In the NC group animals, the reactivity to ACh (10 and 30 nmol) was significantly lower than that observed in naïve rats. Previous treatment with ESCS or ROSU was able to keep these values close to those found in naïve animals. Vascular reactivity to SNP was not significantly altered in any of the experimental groups.

#### 3.4.7. Tissue Redox Status

The effects of prolonged oral treatment with the ESCS (30 and 300 mg/kg) and ROSU on tissue redox status are presented in Table 7. All animals fed with AD and treated only with the vehicle showed a significant increase in LPO levels in the aorta and renal tissues. The prolonged treatment with ESCS (30 and 300 mg/kg) or ROSU maintained these values close to those found in naïve animals. Furthermore, we observed a significant increase in heart, aorta, and kidney SOD activity in animals that were treated with the ESCS (30 and 300 mg/kg) or ROSU. Despite all the treatments carried out raising the tissue SOD activity, ESCS-treatment at a dose of 300 mg/kg induced values statistically higher than those found in all other groups.

#### 3.4.8. Cardiac and Arterial Morphometry

The intimal layer of the subclavian and carotid arteries in the NC group exhibited significant thickening when compared to the naïve group (Table 8). Treatment with the ESCS (30 and 300 mg/kg) or ROSU maintained the thickness of the intima layer of both arteries similarly to naïve rats. The heart ventricles and interventricular septum did not show any significant change between different experimental groups.

#### 3.4.9. Histopathological Analysis

Representative histological images of the liver, heart, and kidney are shown in Figure 3. None of the treatments significantly changed the structure of renal tissue in the different experimental groups. All animals that received the AD exhibited significant lipid accumulation in hepatocytes, with a discrete-to-moderate focus of mononuclear inflammatory infiltrates. The NC group exhibited a diffuse formation of cytoplasmic lipid macro- and microvesicles and slight random individual hepatocyte necrosis. In the ESCS (30 and 300) and ROSU groups, hepatic steatosis was less intense and acquired a microvesicular aspect without displacing the cell nucleus. In cardiac tissue of the NC and ESCS 30 groups, were observed a focal area of fibrous tissue deposition that was associated with mild mononuclear inflammatory infiltrates and cardiac fiber necrosis. This finding was absent in the groups that were treated with ROSU or ESCS 300 mg/kg.

**Figure 4:**
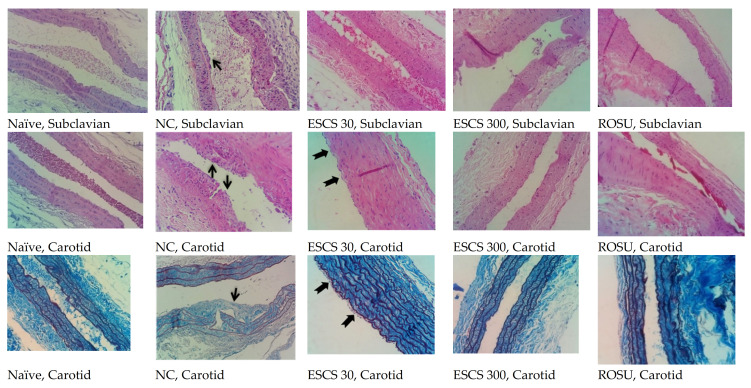
Representative cross-sections of the subclavian and carotid arteries in the naïve, NC, ESCS (30 and 300 mg/kg), and ROSU groups. Black arrows indicate lipid striae. Hematoxylin-eosin (red) or orcein (blue) staining (40×). NC, negative control group; ROSU, rosuvastatin.

Representative histological images of the subclavian and carotid arteries are shown in Figure 4. The NC group exhibited significant atheromatous lesions in the intima layer of the subclavian and carotid arteries. Animals that were treated with ESCS 30 mg/kg exhibited a slight thickening in the intima layer of both arteries, that was compatible with focal lipid streaks. The histopathological pattern of the arteries in the groups that received ROSU or ESCS 300 mg/kg was similar to naïve animals.

## 4. Discussion

A widespread condition in the western population is high levels of blood lipids. In addition to the risk of developing fatty liver disease (FLD), the oxidation of LDL-C particles is a limiting step in the development of atherosclerotic disease [40,41]. The ox-LDL molecules are retained in the inner layer of arteries through a process that is mediated by cytokines that are synthesized by the vascular endothelium, including IL-1β and IL-6 [42]. These cytokines stimulate the expression of adhesion molecules on the endothelial surface, especially VCAM-1, ICAM, and E-selectin [43]. Monocytes then migrate to the subendothelial space and differentiate into macrophages. Macrophages recognize ox-LDL molecules and perform phagocytosis. Finally, filled with lipid inclusions, macrophages become foamy cells that are characteristic of atherosclerotic lesions [44]. 

Several treatments are available around the world to reduce blood lipid levels and the progression of atherosclerotic disease. Among these, statins (e.g., rosuvastatin) deserve to be highlighted. However, side effects (e.g., statin-associated muscle symptoms, diabetes mellitus, and central nervous system complaints) and the high cost of treatment limit its use in some countries [45]. In all major regions of the world, people are looking for additional therapies, including the use of natural products [46]. Tropical countries like Brazil have vast and rich flora, and the use of medicinal plants as an alternative therapy is a very common practice. The present study presents the effectiveness of a natural preparation obtained from *Costus spicatus* leaves and popularly used in the region of Grande Dourados, Mato Grosso do Sul, Brazil [6]. Despite its widespread use, no data to date have indicated any benefits of this species in the treatment of atherosclerosis.

As the first step of this study, the pharmacobotanical features of the leaves of *C. spicatus* were investigated. This investigation provided essential data to identify the species correctly before the preparation of extracts for chemical analyses, besides showing evidence of the main secondary metabolites. These procedures help resolve the adulteration problem, while also contributing to the supply of botanical data, which is still scarce for the species [19,21]. The phytochemical analyses of the ESCS performed by HPLC-DAD-MS resulting in the annotation of 54 compounds. The main compounds identified were flavonoids (flavones and flavonols) and steroidal saponins. Flavonoids are potent antioxidants and are described as therapeutic agents for the treatment of several cardiovascular and inflammatory diseases. These compounds may increase nitric oxide production, decrease ROS and lipid levels, and have antiatherogenic properties [47,48]. Many pharmacological properties also have been described for saponins, such as their ability to reduce lipid and blood glucose, as well as inflammatory effects [49,50]. Although the participation of flavonoids and saponins is an interesting possibility, we believe that the present results are not attributable to the isolated action of a single compound but rather to synergistic and integrated actions of different molecules and multi-target effects [51].

Then, a preclinical trial was performed to evaluate the acute toxicity of ESCS. The administration of ESCS (up to 2000 mg/kg) did not show acute signs of toxicity, indicating a possible pre-clinical safety. With toxicological data showing relative safety, we began the efficacy studies to investigate the possible antiatherosclerotic properties of ESCS. Using a mixed model to induce atherosclerosis in female rats, we induced dyslipidemia, FLD, oxidative stress, and systemic inflammatory response, which contributed to endothelial dysfunction, renal function impairment, and formation of atherosclerotic plaques in different arterial branches. The prolonged treatment with ESCS was able to reduce serum lipids and oxidative stress, increasing SOD activity and reducing lipid peroxidation in renal and arterial tissues. Moreover, we observed a significant reduction of FLD, normalization of endothelial function, and lowest IL-1, IL6, sICAM, and sVCAM levels. Another finding that deserves mention is the diuretic activity induced by ESCS. Although the levels of urinary electrolytes are similar to those obtained in naïve animals, the effects on urinary volume were statically different from control animals, indicating probable solute-free water diuresis. As this response profile can occur with some natural products that act on the hypothalamic-pituitary-adrenal axis [52], the ESCS is likely to have some benefit on renal function regardless of its vascular effects. Together, these effects directly contributed to the maintenance of renal function and a significant reduction in the formation of atherosclerotic plaques in female rats.

Despite the ESCS-highest dose (300 mg/kg) presenting a more expressive response on the formation of focal lipid streaks of the intimate layer of the subclavian and carotid arteries, the data as a whole did not show statistically significant advantages in the other parameters evaluated, not allowing to conclude about a likely dose-dependent effect. The findings presented here provide an important scientific basis about the popular use of *C. spicatus* for the treatment of atherosclerosis. Our data expand scientific knowledge about the species and suggests that the ESCS may be a prototype for a new herbal medicine for the treatment of cardiovascular diseases.

## 5. Conclusions

The data obtained allow us to conclude that the prolonged treatment with ESCS obtained from *Costus spicatus* leaves induce significant hypolipidemic and antioxidant effects, that prevent endothelial dysfunction and modulate the local inflammatory process, reducing the evolution of the atherosclerotic disease.

## Figures and Tables

**Table 1:** Compounds identified from ethanol-soluble fraction of *Costus spicatus* (ESCS) by LC-DAD-MS.

Peak	RT (min)	UV (nm)	MF	Negative Mode (*m/z*)		Positive Mode (*m/z*)		Compound
				MS [M-H]^−^	MS/MS	MS [M+H]^+^	MS/MS	
1	1.1	-	C_5_H_10_O_6_	165.0399	-	-	-	Pentonic acid
2	1.2	-	C_7_H_12_O_8_	223.0449	-	-	-	Unknown
3	1.2	-	C_10_H_19_NO_7_	-	-	266.1234	248, 230, 212, 182, 152	Unknown
4	1.6	-	C_6_H_8_O_7_	191.0201	-	-	-	Citric acid
5	5.4	268, 277, 288	C_11_H_12_N_2_O_2_	203.0822	-	205.0971	188	Tryptophan
6	12.1	299, 325	C_17_H_20_O_11_	399.0924	-	401.1090	-	Caffeic acid derivative
7	14.6	271,334	C_27_H_30_O_15_	593.1509	503, 473, 413, 383, 353	595.1657	505, 475, 439, 421, 409, 391, 379, 355, 325, 295	6,8-Di-*C*-hexosyl apigenin
8	15.9	271, 335	C_26_H_28_O_14_	563.1407	503, 473, 443, 425, 413, 407, 395, 383, 365, 353, 297	565.1568	475, 445, 409, 391, 379, 355, 349, 337, 325, 295	*C*-hexosyl *C*-pentosyl apigenin
9	16.4	271, 335	C_26_H_28_O_14_	563.1397	503, 473, 443, 425, 413, 407, 395, 383, 365, 353	565.1560	475, 445, 409, 391, 379, 355, 349, 337, 325, 307, 295	*C*-hexosyl *C*-pentosyl apigenin
10	16.5	271, 336	C_26_H_28_O_14_	563.1397	485, 473, 455, 443, 425, 413, 407, 395, 383, 365, 353	565.1569	493, 475, 445, 433, 409, 391, 379, 355, 349, 337, 325, 307, 295	*C*-hexosyl *C*-pentosyl apigenin
11	16.7	-	C_11_H_16_O_3_	-	-	197.1179	179, 163, 145.	Unknown
12	17.2	278, 315	C_21_H_41_N_5_O_7_	-	-	476.3095	-	Unknown
13	17.3	272, 338	C_26_H_28_O_14_	563.1399	-	565.1555	475, 445, 391, 379, 355, 337, 325, 307, 295.	*C*-hexosyl *C*-pentosyl apigenin
14	17.6	271, 336	C_26_H_28_O_14_	563.1397	485, 473, 455, 443, 425, 413, 395, 383, 365, 353	565.1573	475, 445, 433, 391, 379, 355, 325, 307, 295	*C*-hexosyl *C*-pentosyl apigenin
15	18	264, 354	C_27_H_30_O_16_	609.1462	300, 271, 255	611.1586	303	O-hexosyl-deoxyhexosyl quercetin
16	18.2	-	C_15_H_20_O_3_	-	-	249.1489	-	Unknown
17	18.3	271, 337	C_27_H_30_O_14_	577.1557	-	579.1706	-	Glycosylated flavone
18	18.5	262, 352	C_27_H_30_O_16_	609.1444	300, 285, 271, 255, 243, 179	611.1619	465, 303	Rutin^*^
19	18.8	262, 356	C_21_H_20_O_12_	463.0871	300, 271, 255, 243, 227, 179	465.1051	303	*O*-hexosyl quercetin
20	19.2	270, 338	C_27_H_30_O_14_	577.1514	-	579.1728	507, 489, 459, 429, 423, 405, 379, 355, 359, 337, 325, 271	*C*-hexosyl *C*-deoxyhexosyl apigenin
21	19.3	266, 343	C_27_H_30_O_15_	593.1490	447, 429, 284, 255, 227	595.1639	449, 287	O-deoxyhexosyl-hexosyl luteolin
22	19.5	265, 341	C_27_H_30_O_15_	593.1494	284, 255, 227, 151	595.1675	287	O-deoxyhexosyl-hexosyl luteolin
23	19.5	265, 350	C_20_H_18_O_11_	433.0767	300, 283, 271, 255, 243, 227, 179	435.0926	303	*O*-pentosyl quercetin
24	20.0	263, 353	C_20_H_18_O_11_	433.0757	300, 271, 255, 243, 227, 179	435.0936	303	*O*-pentosyl quercetin
25	20.6	263, 340	C_27_H_30_O_15_	593.1494	285, 284, 255, 239, 229, 227	595.1650	449, 287	*O*-hexosyl-deoxyhexosyl luteolin
26	20.8	264, 350	C_28_H_32_O_16_	623.1596	314, 299, 287, 285, 271, 243	625.1747	479, 317	*O*-hexosyl-deoxyhexosyl *O*-methylquercetin
27	20.9	265, 341	C_21_H_20_O_11_	447.0915	285, 255, 227, 211	449.1073	287, 261, 213, 153	*O*-hexosyl luteolin
28	21.0	264, 348	C_21_H_20_O_11_	447.0915	300, 271, 255, 227, 179	449.1077	303, 257, 229, 153	*O*-deoxyhexosyl quercetin
29	21.2	262, 349	C_22_H_22_O_12_	477.1044	314, 300, 285, 271, 257, 243, 227	479.1134	317, 302, 285, 274, 257, 246, 228, 153	*O*-hexosyl *O*-methyl quercetin
30	21.3	264, 352	C_28_H_32_O_16_	623.1606	315, 299, 287, 269, 255, 243	625.1768	317, 302	*O*-hexosyl-deoxyhexosyl *O*-methyl quercetin
31	21.5	265, 342	C_20_H_18_O_10_	417.0817	284, 255, 227	419.0969	287, 268, 258, 231, 213, 165,153	*O*-pentosyl luteolin
32	21.5	265, 346	C_26_H_28_O_14_	563.1399	284, 255, 227	565.1553	287, 165, 153	*O*-pentosyl-deoxyhexosyl luteolin
33	21.6	262, 352	C_22_H_22_O_12_	477.1026	314, 299, 285, 271, 257, 243, 227, 175	479.1198	317, 302, 285, 274, 257, 229, 153	*O*-hexosyl *O*-methylquercetin
34	22	265, 343	C_20_H_18_O_10_	417.0818	-	419.1980	287	*O*-pentosyl luteolin
35	22.9	269, 352	C_21_H_20_O_11_	447.0924	-	449.1063	317	*O*-pentosyl *O*-methylquercetin
36	23.1	267, 350	C_22_H_22_O_11_	461.1072	446, 299, 283, 255, 227, 211, 199, 183, 185	463.1247	301, 286, 258	*O*-hexosyl *O*-methyl kaempferol
37	23.5	265, 340	C_21_H_20_O_10_	431.0973	284, 255, 227	433.140	287	*O*-deoxyhexosyl luteolin
38	24.9	295, 322	C_11_H_12_O_4_	207.0673	161, 179	209.0810	163	Di-*O*-methyl caffeic acid
39	28.8	299, 315	C_28_H_38_O_10_	533.2409	189, 179, 163, 161, 145	535.2547	-	Unknown
40	29.2	299, 325	C_29_H_40_O_11_	563.2509	548, 251, 221, 207, 193, 189, 175, 160	565.2632	-	Unknown
41	29.6	-	C_51_H_84_O_22_	1047.5367	901, 883, 755, 247	1031.5444 [M+H-H_2_O]^+^	869, 415, 397, 379, 293, 271, 253	Steroidal saponin(di-*O*-hexosyl di-*O*-deoxyhexosyl)
42	29.7	-	C_50_H_82_O_22_	1033.5195	901, 887, 755, 233	1017.5296 [M+H-H_2_O]^+^	855, 739, 711, 415, 397, 379, 309, 271, 253	Steroidal saponin (di-*O*-hexosyl *O*-pentosyl *O*-deoxyhexosyl)
43	30.2	280, 318	C_18_H_32_O_5_	327.2183	239, 229, 221, 211, 183, 171, 165	351.2149 [M+Na]^+^	-	Unknown
44	30.3	268, 348	C_16_H_12_O_6_	299.0567	284, 255, 227, 211, 199, 185	301.0707	286, 269, 258, 241, 229, 213, 193, 188, 184, 153.	*O*-Methyl kaempferol
45	30.3	279, 321	C_18_H_32_O_5_	327.2189	239, 229, 221, 211, 197, 193, 185, 183, 171	351.2136 [M+Na]^+^	-	Unknown
46	30.6	280, 325	C_18_H_32_O_5_	327.2190	259, 229, 211, 199, 183, 171.	351.2142 [M+Na]^+^	-	Unknown
47	30.6	-	C_44_H_74_O_17_	873.4888	873, 741, 595, 433, 379, 349, 289, 275, 247, 233, 205, 191, 179, 163	875.5024	743	Steroidal saponin (*O*-pentosyl *O*-deoxyhexosyl *O*-hexosyl)
48	30.7	-	C_39_H_66_O_13_	741.4405	741, 595, 433, 415, 325, 287, 205, 179, 163, 143	743.4577	-	Steroidal saponin (*O*-deoxyhexosyl *O*-hexosyl)
49	31.1	-	C_38_H_64_O_13_	727.4304	727, 595, 433, 251, 233, 191, 179, 161.	729.4429		Steroidal saponin (*O*-pentosyl *O*-hexosyl)
50	32.3	-	C_18_H_34_O_5_	329.2329	229, 211, 193, 183, 171	353.2298 [M+Na]^+^		Fatty acid
51	32.4	-	C_18_H_16_O_7_	343.0821	-	345.0979	330, 312, 284, 269, 256, 241, 149	Unknown
52	34.6	-	C_18_H_30_O_4_	309.2079	197, 179, 169	333.2033 [M+Na]^+^	-	Unknown
53	34.8	-	C_18_H_30_O_4_	309.2084	211, 197, 183, 169	333.2041 [M+Na]^+^	-	Unknown
54	35.7	282, 321	C_33_H_56_O_14_	675.3568	415, 397, 379, 305, 287, 277, 263, 253, 235, 221, 179, 161	699.3586 [M+Na]^+^	537	Unknown

RT: retention time; MF: molecular formula. All the MF was determined from the errors and mSigma below 5 ppm and 30, respectively.

**Table 2:** Effects of treatment with ESCS (30 and 300 mg/kg) and rosuvastatin (ROSU) on urinary parameters of female rats treated with atherogenic diet or standard feed.

Parameter	Naïve	NC	ESCS (30 mg/kg)	ESCS (300 mg/kg)	ROSU (5 mg/kg)
Urinary volume (mL/100 g/24 h)	8.67 ± 0.56	8.48 ± 0.65	14.07 ± 0.82 ^a,b,c^	15.72 ± 0.97 ^a,b,c^	9.20 ± 0.67
pH	7.60 ± 0.19	6.34 ± 0.15 ^a^	7.09 ± 0.15 ^b^	7.37 ± 0.07 ^b^	7.11 ± 0.16 ^b^
Density	1033 ± 2.24	1020 ± 1.94 ^a^	1030 ± 0.98 ^b^	1031 ± 0.68 ^b^	1030 ± 2.06 ^b^
Chloride (µmol/100 g/24 h)	953 ± 112.70	526.20 ± 87.30 ^a^	898.74 ± 98.69 ^b^	970.70 ± 88.86 ^b^	889.80 ± 81.32 ^b^
Magnesium (mg/100 g/24 h)	1.36 ± 0.28	2.27 ± 0.57	1.54 ± 0.43	1.15 ± 0.38	0.47 ± 0.23
Potassium (µmol/100 g/24 h)	1275 ± 139.80	988.90 ± 159.20	941.40 ± 84.28	881.10 ± 71.03	829.20 ± 48.69
Sodium (µmol/100 g/24 h)	641.80 ± 93.46	608.90 ± 128	413.50 ± 35.03	399.90 ± 25.08	406.10 ± 25.46
Calcium (mg/100 g/24 h)	1.49 ± 0.13	5.94 ± 2.49	4.27 ± 0.79	3.52 ± 0.55	3.58 ± 0.25
Creatinine (mg/100 g/24 h)	2.97 ± 0.18	3.73 ± 0.39	3.52 ± 0.11	3.33 ± 0.14	3.15 ± 0.04

Statistical analyses were performed using one–way ANOVA followed by the Bonferroni post hoc test. Values are expressed as mean ± SEM (standard error of the mean). ^a^ *p* ≤ 0.05 when compared to naïve; ^b^ *p* ≤ 0.05 when compared to NC group; ^c^ *p* ≤ 0.05 when compared to ROSU group. ESCS: animals treated with ethanol-soluble fraction of *Costus spicatus* (30 or 300 mg/kg) and fed with an atherogenic diet; Naïve: animals treated with vehicle (water) and fed with a standard diet; NC: animals treated with vehicle (water) and fed with an atherogenic diet; ROSU: animals treated with rosuvastatin (5 mg/kg) and fed with an atherogenic diet.

**Table 3:** Effects of treatment with ESCS (30 and 300 mg/kg) and rosuvastatin (ROSU) on electrocardiographic parameters of female rats treated with atherogenic diet or standard feed.

	Naïve	NC	ESCS (30 mg/kg)	ESCS (300 mg/kg)	ROSU (5 mg/kg)
*Segments (ms)*					
PR	46.47 ± 2.72	42.59 ± 1.59	40.83 ± 4.64	35.50 ± 4.95	41.75 ± 3.70
QRS	37.89 ± 1.37	39.57 ± 1.67	43.60 ± 2.11	42.67 ± 1.66	40.20 ± 1.02
QT	134.10 ± 7.66	131.60 ± 9.41	120.80 ± 6.99	115.20 ± 10.86	121.40 ± 12.24
QTC	232.70 ± 9.89	234.90 ± 15.69	240 ± 20.84	197 ± 22.95	225.40 ± 23.94
*Waves (mV)*					
P	0.083 ± 0.004	0.063 ± 0.003	0.052 ± 0.019	0.053 ± 0.018	0.046 ± 0.006
Q	−0.007 ± 0.002	−0.011 ± 0.002	−0.025 ± 0.007	−0.020 ± 0.008	−0.012 ± 0.010
R	0.374 ± 0.016	0.382 ± 0.023	0.295 ± 0.040	0.330 ± 0.049	0.310 ± 0.027
S	0.052 ± 0.017	0.014 ± 0.018	0.026 ± 0.007	−0.006 ± 0.010	0.006 ± 0.018

Statistical analyses were performed using one–way ANOVA followed by the Bonferroni *post hoc* test. Values are expressed as mean ± SEM (standard error of the mean). ESCS: animals treated with ethanol-soluble fraction of *Costus spicatus* (30 or 300 mg/kg) and fed with an atherogenic diet; Naïve: animals treated with vehicle (water) and fed with a standard diet; NC: animals treated with vehicle (water) and fed with an atherogenic diet; ROSU: animals treated with rosuvastatin (5 mg/kg) and fed with an atherogenic diet.

**Table 4:** Effects of treatment with ESCS (30 and 300 mg/kg) and rosuvastatin (ROSU) on blood pressure and heart rate values of female rats treated with atherogenic diet or standard feed.

Parameter	Naïve	NC	ESCS (30 mg/kg)	ESCS (300 mg/kg)	ROSU (5 mg/kg)
DBP (mm Hg)	57.23 ± 5.76	56.08 ± 7.89	60.23 ± 4.18	69.90 ± 7.66	64.83 ± 4.29
SBP (mm Hg)	89.93 ± 6.70	98.55 ± 10.61	95.48 ± 6.89	104.90 ± 9.67	100.40 ± 9.01
MAP (mm Hg)	74.46 ± 6.06	76.94 ± 9.39	78.90 ± 5.83	89.25 ± 8.06	83.38 ± 6.23
HR (bpm)	192.40 ± 18.30	228.60 ± 21.57	215.60 ± 18.23	257.60 ± 33	201.60 ± 17.83

Statistical analyses were performed using one–way ANOVA followed by the Bonferroni *post hoc* test. Values are expressed as mean ± SEM (standard error of the mean). bpm: beats per minute; DBP: diastolic blood pressure; ESCS: animals treated with ethanol-soluble fraction of *Costus spicatus* (30 or 300 mg/kg) and fed with an atherogenic diet; HR: heart rate; MAP: mean arterial pressure; Naïve: animals treated with vehicle (water) and fed with a standard diet; NC: animals treated with vehicle (water) and fed with an atherogenic diet; ROSU: animals treated with rosuvastatin (5 mg/kg) and fed with an atherogenic diet; SBP: systolic blood pressure.

**Table 5:** Effects of treatment with ESCS (30 and 300 mg/kg) and rosuvastatin (ROSU) on biochemical parameters of female rats treated with atherogenic diet or standard feed.

Parameter	Naïve	NC	ESCS (30 mg/kg)	ESCS (300 mg/kg)	ROSU (5 mg/kg)
AST (U/L)	171.83 ± 43.21	120.70 ± 46.02	117.20 ± 11.25	123.97 ± 12.71	118.14 ± 17.35
ALT (U/L)	42.77 ± 6.22	46.18 ± 5.51	43.88 ± 7.80	35.38 ± 7.92	48.56 ± 6.98
TG (mg/dL)	69.12 ± 7.11	156.11 ± 10.03 ^a^	80.48 ± 8.77 ^b^	77.94 ± 9.37 ^b^	88.25 ± 10.25 ^b^
TC (mg/dL)	71.37 ± 14.02	168.14 ± 9.13 ^a^	121.11 ± 6.63 ^a,b^	109.18 ± 6.18 ^a,b^	114.06 ± 8.30 ^a,b^
HDL-C (mg/dL)	27.12 ± 5.04	40.21 ± 8.21	56.44 ± 5.15 ^a^	59.05 ± 8.27 ^a^	63.12 ± 6.78 ^a^
VLDL-C (mg/dL)	13.82 ± 3.22	33.63 ± 7.71 ^a^	8.09 ± 1.13 ^b^	7.98 ± 0.27 ^b^	5.01 ± 0.85 ^b^
LDL-C (mg/dL)	20.01 ± 2.33	96.80 ± 9.32 ^a^	66.60 ± 7.96 ^a,b^	56.14 ± 5.47 ^ab^	55.94 ± 6.58 ^a,b^
oxLDL (ng/mL)	0.20 ± 0.05	2.52 ± 0.08 ^a^	1.82 ± 0.12 ^ab^	1.12 ± 0.11 ^a,b^	0.90 ± 0.04 ^a,b^
sVCAM-1 (ng/L)	2.21 ± 0.04	6.43 ± 0.30 ^a^	4.66 ± 0.11 ^ab^	4.10 ± 0.18 ^a,b^	4.60 ± 0.12 ^a,b^
sICAM-1 (ng/L)	4.70 ± 0.32	12.16 ± 0.77 ^a^	8.12 ± 0.62 ^ab^	7.60 ± 0.73 ^a,b^	6.01 ± 0.24 ^a,b^
IL-6 (ng/L)	156.15 ± 11.2	298.58 ± 22.1 ^a^	233.12 ± 21.2 ^a,b^	214.23 ± 19.3 ^a,b^	215.21 ± 22.2 ^a,b^
IL-1β (pg/mL)	311.11 ± 23.2	654.33 ± 22.2 ^a^	480.22 ± 19.1 ^a,b^	441.17 ± 25.1 ^a,b^	400.35 ± 20.3 ^a,b^
Creatinine (mg/dL)	0.38 ± 0.09	0.36 ± 0.06	0.42 ± 0.02	0.44 ± 0.08	0.38 ± 0.02
Urea (mg/dL)	32.06 ± 2.34	37.02 ± 10.09	34.65 ± 4.39	36.34 ± 5.76	38.18 ± 2.08

Statistical analyses were performed using one-way ANOVA followed by the Bonferroni *post hoc* test. Values are expressed as mean ± SEM (standard error of the mean). ^a^ *p* ≤ 0.05 when compared to naïve; ^b^ *p* ≤ 0.05 when compared to NC group. **ALT:** alanine aminotransferase; AST: aspartate aminotransferase; ESCS: animals treated with ethanol-soluble fraction of *Costus spicatus* (30 or 300 mg/kg) and fed with an atherogenic diet; HDL-C: high-density lipoprotein cholesterol; IL-6: interleukin-6; IL-1β: interleukin-1β; LDL-C: low-density lipoprotein cholesterol; Naïve: animals treated with vehicle (water) and fed with a standard diet; NC: animals treated with vehicle (water) and fed with an atherogenic diet; oxLDL: oxidized low-density lipoprotein; ROSU: animals treated with rosuvastatin (5 mg/kg) and fed with an atherogenic diet; sICAM: soluble intercellular adhesion molecule-1; sVCAM-1: soluble vascular cell adhesion molecule-1; TC: total cholesterol; TG: triglycerides; VLDL-C: very-low-density lipoprotein cholesterol.

**Table 6:** Effects of treatment with ESCS (30 and 300 mg/kg) and rosuvastatin (ROSU) on changes of perfusion pressure (mm Hg) obtained in mesenteric vascular beds of female rats treated with atherogenic diet or standard feed.

Drug (Dose)	Naïve	NC	ESCS (30 mg/kg)	ESCS (300 mg/kg)	ROSU (5 mg/kg)
*Phe (nmol)*					
1	2.150 ± 1.327	0.742 ± 0.261	0.481 ± 0.574	0.265 ± 0.558	0.828 ± 0.447
3	1.054 ± 0.345	0.896 ± 0.319	0.841 ± 0.575	1.270 ± 0.786	0.892 ± 0.407
10	1.757 ± 0.575	4.082 ± 0.415 ^a^	2.501 ± 0.468 ^b^	2.647 ± 0.454 ^b^	2.833 ± 0.396 ^b^
30	8.075 ± 1.475	13.769 ± 1.427 ^a^	5.737 ± 2.667 ^b^	5.148 ± 2.360 ^b^	15.970 ± 3.579 ^a,c^
*ACh (pmol)*					
1	−3.403 ± 1.644	−2.471 ± 0.876	−4.467 ± 6.791	−2.821 ± 0.751	2.302 ± 5.947
3	−4.247 ± 2.190	−3.073 ± 1.902	−2.094 ± 2.983	−3.194 ± 1.153	−3.113 ± 0.944
10	−6.159 ± 1.376	−2.919 ± 1.029 ^a^	−4.907 ± 1.437	−5.119 ± 1.188	−5.532 ± 1.712
30	−9.194 ± 1.269	−4.383 ± 1.226 ^a^	−6.424 ± 2.233	−6.665 ± 1.897	−5.820 ± 2.142
*SNP (pmol)*					
0.1	−2.170 ± 1.135	−2.114 ± 0.972	1.692 ± 0.796	0.768 ± 0.916	−0.885 ± 0.961
0.3	−3.054 ± 0.814	−4.402 ± 1.762	−1.889 ± 1.219	−3.042 ± 1.369	−3.129 ± 12.067
1	−4.395 ± 1.380	−6.660 ± 3.275	−5.711 ± 0.763	−4.262 ± 2.384	−4.286 ± 1.034
3	−4.571 ± 2.987	−4.402 ± 1.762	−5.697 ± 4.579	−5.045 ± 3.250	−4.336 ± 2.074

Statistical analyses were performed using one-way ANOVA followed by the Bonferroni *post hoc* test. Values are expressed as mean ± SEM (standard error of the mean). ^a^ *p* ≤ 0.05 when compared to naïve; ^b^ *p* ≤ 0.05 when compared to NC group; ^c^ *p* ≤ 0.05 when compared to ESCS (30 and 300 mg/kg) group. ACh: acetylcholine; ESCS: animals treated with ethanol-soluble fraction of *Costus spicatus* (30 or 300 mg/kg); Naïve: animals treated with vehicle (water) and fed with a standard diet; NC: animals treated with vehicle (water) and fed with an atherogenic diet; Phe: phenylephrine; ROSU: animals treated with rosuvastatin (5 mg/kg) and fed with an atherogenic diet; SNP: sodium nitroprusside.

**Table 7:** Effects of treatment with ESCS (30 and 300 mg/kg) and rosuvastatin (ROSU) on tissue redox status of female rats treated with atherogenic diet or standard feed.

Parameter	Naïve	NC	ESCS (30 mg/kg)	ESCS (300 mg/kg)	ROSU (5 mg/kg)
*Heart*					
SOD (unit of SOD/g tissue)	25.67 ± 1.18	27.92 ± 1.90	71.88 ± 2.31 ^a,b^	123.30 ± 4.78 ^a,b,c^	74.06 ± 2.15 ^a,b^
LPO (nmol hydroperoxides/g tissue)	225. 40 ± 77.10	219.01 ± 46.36	222.50 ± 16.20	212.30 ± 12.57	223.50 ± 16.45
*Aorta*					
SOD (unit of SOD/g tissue)	22.62 ± 0.81	22.41 ± 1.85	53.39 ± 3.61 ^a,b^	92.37 ± 2.05 ^a,b,c^	44.33 ± 1.37 ^a,b^
LPO (nmol hydroperoxides/g tissue)	145.20 ± 27.08	439.20 ± 34.41 ^a^	181.10 ± 19.34 ^b^	185.9 ± 26.72 ^b^	188.70 ± 28.10 ^b^
*Kidney*					
SOD (unit of SOD/g tissue)	18.17 ± 4.54	24.59 ± 6.38	103.90 ± 6.41 ^a,b^	132.30 ± 6.70 ^a,b,c^	90.11 ± 5.17 ^a,b^
LPO (nmol hydroperoxides/g tissue)	108.40 ± 17.53	262.90 ± 26.16 ^a^	147.20 ± 21.19 ^b^	151.90 ± 27.84 ^b^	148.30 ± 23.67 ^b^

Statistical analyses were performed using one-way ANOVA followed by the Bonferroni *post hoc* test. Values are expressed as mean ± SEM (standard error of the mean). ^a^ *p* ≤ 0.05 when compared to naïve; ^b^ *p* ≤ 0.05 when compared to NC group; ^c^ *p* ≤ 0.05 when compared to ROSU group. ESCS: animals treated with ethanol-soluble fraction of *Costus spicatus* (30 or 300 mg/kg) and fed with an atherogenic diet; LPO: lipid peroxidation; Naïve: animals treated with vehicle (water) and fed with a standard diet; NC: animals treated with vehicle (water) and fed with an atherogenic diet; ROSU: animals treated with rosuvastatin (5 mg/kg) and fed with an atherogenic diet; SOD: superoxide dismutase.

**Table 8:** Effects of treatment with ESCS (30 and 300 mg/kg) and rosuvastatin (ROSU) on cardiac and arterial morphometry of female rats treated with atherogenic diet or standard feed.

Parameter	Naïve	NC	ESCS (30 mg/kg)	ESCS (300 mg/kg)	ROSU (5 mg/kg)
Right ventricle (µm)	365.60 ± 4.32	345.90 ± 27.53	395.90 ± 33.17	362.60 ± 28.85	402.60 ± 32.09
Left ventricle (µm)	757.70 ± 76.72	629.60 ± 76.90	638.50 ± 60	658.30 ± 56.36	740.5 ± 44.70
IV septum (µm)	527.21 ± 49.39	424.34 ± 47.20	435.70 ± 36.47	515.20 ± 26.92	523.50 ± 44.41
SA (intima layer; µm)	3.78 ± 0.57	64.80 ± 3.67 ^a^	5.11 ± 0.59 ^b^	4.64 ± 0.39 ^b^	5.06 ± 0.67 ^b^
CA (intima layer; µm)	2.95 ± 0.29	29.61 ± 2.49 ^a^	5.02 ± 0.89 ^b^	3.36 ± 0.48 ^b^	2.51 ± 0.25 ^b^

Statistical analyses were performed using one-way ANOVA followed by the Bonferroni *post hoc* test. Values are expressed as mean ± SEM (standard error of the mean). ^a^ *p* ≤ 0.05 when compared to naïve; ^b^ *p* ≤ 0.05 when compared to NC group. CA: carotid artery; ESCS: animals treated with ethanol-soluble fraction of *Costus spicatus* (30 or 300 mg/kg) and fed with an atherogenic diet; IV: interventricular septum; Naïve: animals treated with vehicle (water) and fed with a standard diet; NC: animals treated with vehicle (water) and fed with an atherogenic diet; ROSU: animals treated with rosuvastatin (5 mg/kg) and fed with an atherogenic diet; SA: subclavian artery.
